# Are Consanguineous Marriages to Blame for Usher Syndrome Type 1, a Rare Disease in Pakistan?

**DOI:** 10.7759/cureus.11117

**Published:** 2020-10-23

**Authors:** Ali I Awan, Eusha Abdul Raffay, Ayesha Liaqat, Taimoor Hassan, Maria Khan

**Affiliations:** 1 Psychiatry and Behavioral Sciences, King Edward Medical University, Mayo Hospital, Lahore, PAK; 2 Internal Medicine, Services Institute of Medical Sciences, Lahore, PAK; 3 Internal Medicine, District Headquarter Hospital, Nankana Sahib, PAK; 4 Internal Medicine, King Edward Medical University, Mayo Hospital, Lahore, PAK

**Keywords:** autosomal recessive, sensorineural hearing loss, retinitis pigmentosa, case report, consanguineous marriages, usher syndrome, genetical counselling, pakistan

## Abstract

Usher syndrome type I is a rare genetic autosomal recessive disease caused by mutations in specific genes that provide instructions for making proteins involved in normal hearing, vision, and balance. It is characterized by hearing impairment due to the inability of auditory nerves to send sensory input to the brain leading to hearing loss along with retinitis pigmentosa (RP), which is a progressive, bilateral, symmetrical retinal degeneration involving photoreceptor cells. We report a 32-year-old male patient who presented to us with complaints of night blindness and progressive vision loss for the past 20 years. He had bilateral hearing loss leading to deaf-mutism. In addition, his developmental milestones were delayed. His fundoscopic findings were consistent with RP and his electroretinography confirmed reduced retinal activity. Pure tone audiometry confirmed bilateral sensory neural hearing. His mother was a known case of Usher syndrome type 1. His family history was remarkable for multiple consanguineous marriages in both his parental and maternal families and a confirmed diagnosis of Usher syndrome in paternal uncle. The patient was tried on hearing aids and vitamin A medication but with minimal improvement in his overall condition. A multidisciplinary approach, involving an audiologist, speech, and language therapist was adapted to help the patient. Early genetic testing can help diagnose such cases in its early stages and genetic counseling regarding the detrimental effects of consanguineous marriages can play a very positive role in genetic diseases, especially those with autosomal recessive inheritance patterns.

## Introduction

Usher syndrome type 1 is characterized by the combination of retinitis pigmentosa (RP), profound congenital deafness, and vestibular ataxia [1]. The syndrome is transmitted by the autosomal recessive mode and currently there are six genes associated with this syndrome: Myosin VIIA (MYO7A), Usher syndrome 1C (USH1C), Cadherin-23 (CDH23), Protocadherin−15 (PCDH15), Usher syndrome 1G, and Calcium and integrin-binding family member 2 [1]. The mutated proteins are actually part of a dynamic protein complex that is present in hair cells of the inner ear i.e., cochlea and in photoreceptors of the retina. This Usher protein complex is essential in the formation of the stereo cilia bundle in hair cells and in the calycal processes of photoreceptor cells [2]. Usher syndrome has been divided into three basic types, USH1, USH2 and USH3 with subtypes denoted as USH1a and USH1b, however, a fourth type of usher syndrome has also been suggested called atypical US, in which there are late-onset RP and hearing loss without vestibular involvement. In the population aged over 15 years the prevalence was 6.2 per 100,000 population for all US subtypes [3]. There is no evidence-based data available on the prevalence of this disease in Pakistan, which in fact shows the rarity of this disease as well as the need to explore the challenges involved in the diagnosis, investigations, management, and reporting of the disease in Pakistan. 

## Case presentation

A single 32-year-old male patient of Pakistani ethnicity presented in the Ophthalmology Department of Services Hospital, Lahore with the complaint of night blindness for the past 20 years. His vision was normal during his childhood years but it started deteriorating after some time. There was no history of pain, redness, discharge, or photophobia. The patient had a history of bilateral cataract extraction but he again developed the complaint of cloudy vision caused by one of the complications of cataract surgery i.e. opacification of the lens capsule. He was referred to the Services Hospital where his yttrium aluminum garnet (YAG) capsulotomy was performed. On further history taking it was revealed that the patient was congenitally deaf leading to deaf-mutism. His developmental milestones were delayed up to 18 months of age. He also had balance issues and needed support to walk. 

His family history revealed that his mother had bilateral hearing loss with poor vision since childhood. She was a diagnosed case of Usher syndrome type I which was confirmed when we asked for her genetic and clinical reports. Her pure tone audiometry report showed profound bilateral sensorineural hearing loss. Her eye examination reports showed RP which is consistent with the diagnosis. Her visual acuity was 2/60 for the right eye and 2/60 for the left eye. His father was phenotypically normal. It was also found that the parents and the grandparents of the affected individual had consanguineous marriages. Both the grandparents were phenotypically normal. One of the paternal uncles had decreased vision with complete sensorineural hearing loss and was a diagnosed case of Usher syndrome which further strengthened the diagnosis of Usher syndrome in our patient. After investigating the patient’s medical history, we proceeded towards clinical examination. 

**Figure 1:**
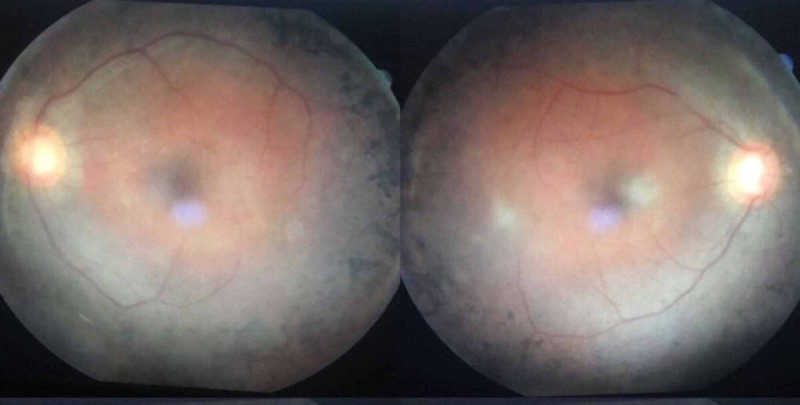
Retinitis pigmentosa in a patient with Usher Syndrome type I

On examination of the eyes of our patient, he had a visual acuity of 6/9 of both eyes with no improvement by pinhole. His extraocular movements were normal with negative relative afferent pupillary defect (RAPD). On slit-lamp examination, external examination was unremarkable with clear cornea and sclera, bilateral pseudophakia was present. Intraocular pressure was normal. On ophthalmoscopy, fundus showed pale optic disc, attenuated blood vessels and bony spicules (clumps of retinal pigment epithelium). In order to make the absolute diagnosis, electroretinography was performed to measure the electrical activity of the retina which revealed exactly what we were suspecting i.e., reduced electrical activity of the retina indicating that photoreceptors were not functioning properly, which is a key feature of Usher syndrome type I. Fundoscopy findings can be seen in Figure 1.

**Figure 2:**
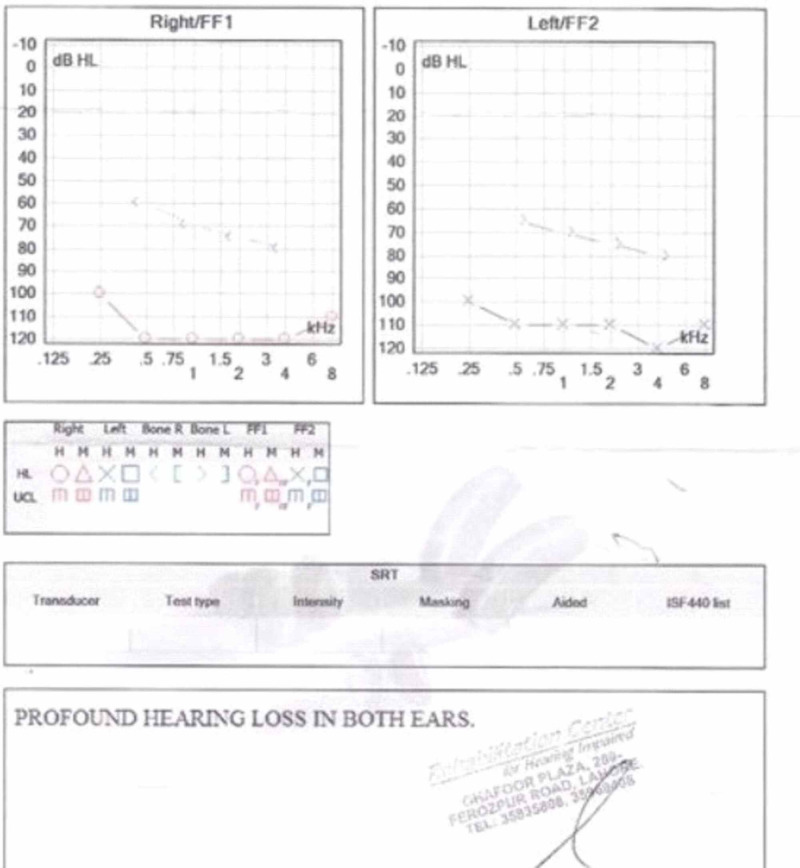
Audiometry report showing profound sensory neural hearing loss (SNHL) in a patient with Usher syndrome type I

After examination of the eyes, pure tone audiometry was performed indicating profound sensory neural hearing loss (SNHL) in both ears i.e. represented by values below 90dB on graph. His systemic examination was normal overall except distal muscle weakness. Blood reports revealed an increased level of creatinine kinase (CK), showing some degree of ongoing myopathy. His X-ray indicated no lymphadenopathy and ultrasound showed normal liver and kidney, ruling out Alport syndrome. His echocardiography was also normal, which ruled out the possibility of congenital Rubella syndrome. On the basis of the aforementioned findings, a definitive diagnosis of Usher syndrome type 1 was established. Testing for specific chromosomal abnormalities was not performed due to limited resources, which is one of the major challenges faced in diagnosing rare genetical disorders in a developing country like Pakistan. Audiometry findings can be seen in Figure 2.

Once the diagnosis was established, a multidisciplinary approach was adapted in order to manage this patient. He was given a trial of hearing aids by the Ear, Nose & Throat (ENT) department but due to extent of the cochlear involvement there was no significant improvement. For his vision, he was given a trial of vitamin A palmitate supplementation with careful evaluation of his liver function test (LFTS) because of its hepatotoxicity effects with the dosage not exceeding 15000 IU per day in order to limit the retinitis pigmentosa. The trial lasted for 15 days with no betterment in vision or decline in severity of RP. Genetic counseling was done as the patient was unmarried and had a history of consanguineous marriage among his parents and grandparents. The patient was referred to a speech and language therapist along with monthly evaluation by an audiologist, as such patients are good candidates for cochlear implantation a long-term plan included a possibility of cochlear implant. Follow-up visits along with psychiatric help was offered to the patient and family members. 

## Discussion

In Usher syndrome type I, there is congenital sensorineural hearing loss, vestibular areflexia and progressive peripheral vision loss due to retinitis pigmentosa which is not present at birth but manifests later in the course of disease. Individual affected by USH1 walks later than expected usually at 18 months or it can take up to two years of age, due to vestibular ataxia. These children are usually misdiagnosed, especially in Pakistan. This is partly because of the rarity of the disease and partly because of the lack of facilities to carry out genetic analysis. 

Usher syndrome type I is a genetic disorder inherited as an autosomal recessive trait. It is caused when each of the carrier parent has the same mutated usher gene. When both parents are asymptomatic carriers of the Usher syndrome gene, they have a 25% chance with each pregnancy of producing a child with Usher syndrome [4]. It has been proven by studies that the occurrence of an autosomal recessive trait is common with consanguineous marriages. The offspring of consanguineous couples are at increased risk for autosomal recessive disorders due to their increased risk for homozygosity by descent [5]. Consanguineous marriages have always been a problem in South Asian and Middle Eastern countries. Around 70% of marriages in Pakistan are consanguineous [6]. One of the major reasons for these increased number of consanguineous marriages is the lack of awareness about the genetic pathologies and associated diseases. 

In addition, these children, with rare genetic disorders that need extensive evaluation and molecular diagnostic testing, are usually misdiagnosed. However, recent studies have shown that DNA testing for Usher syndrome is feasible, and that it could potentially be incorporated into the newborn screening program [7]. Early diagnosis would have positive implications for educational planning, genetic counseling, and treatment [8].

## Conclusions

Considering the challenges involved in the diagnosis and management of rare genetic disorders as seen in this patient, it is crucial to highlight the importance of genetic counseling regarding consanguineous marriages. Genetic counseling should be done in the families in which this disease runs so that they are aware of the complications and risk factors associated with it. Management of such patients can be done by the use of hearing aids, assistive listening devices, mobility training, cochlear implants, and low vision services, and research on the effectiveness of such treatment modalities for this disease should be done.
